# Death of Father Kneipp

**Published:** 1897-08

**Authors:** 


### Editorial.

Drs. W. S. Elkin and Hunter P. Cooper will open their private sanitorium about October 1.

The building is of granite and brick, four stories high, and contains fifty-two rooms. The building is fitted throughout with all the modern conveniences and has been constructed with a close regard to sanitary and hygienic requirements.

The professional reputation and standing of these gentlemen is already so well known as to require no indorsement from us. We feel sure that any patient entrusted to their care will receive the benefit of science and skill and complete hospital equipment, conscientiously carried out.

Atlanta has now, besides the Grady Hospital and St. Joseph's Infirmary, Dr. Holmes' Sanitorium and the Atlanta Retreat conducted by Dr. C. C. Stockard, both admirably equipped and managed, the latter being particularly intended for medical cases and the treatment of drug habits and alcoholism.

The institutions of Dr. Holmes and Drs. Cooper and Elkin are especially designed for the reception and treatment of general surgical and orthopedic cases among patients who reside in town, but who find it inexpedient to be treated at their homes and particularly for out-of-town patients, who desire to avail themselves of the best surgical advice and assistance. To all such, these sanitoriums can be unreservedly recommended.

### DEATH OF FATHER KNEIPP.

Father Sebastian Kneipp, the genial old priest whose water cure, or grass cure, made him famous, died at Woerishofen, Bavaria, on June 17, in the seventy-sixth year of his age. Father Kneipp was a unique figure in the history of the healing art. His fame came from his original method of treating diseased persons by means, chiefly, of cold water applied in a variety of ways. He practiced the cure for over a lifetime, although it came into general vogue only in the last five years. He was born in 1821, and after leaving school worked as a weaver until the age of twenty-seven, when he began to study medicine and theology, having long desired to become a priest. He was in ill health, and in a delirium of fever he rushed from his room and thrust his feet through the ice in a pond, and instead of becoming worse found he was much better for the shock, and so began systematic experiments along this line.

He was admitted to holy orders and went to the village of Woerishofen, in Bavaria, where he earned the love of his neighbors and the mountain folk, whom he had cured of disease by the cold water treatment. His fame was for a long time local, but in time it spread all over the world, and people came to him for treatment in large numbers. The doctors looked askance at the spectacle of a priest making use of the methods only ascribed to a charlatan, but he really was no charlatan. At last notable persons began to come to him for treatment. Emperor Francis Joseph took a course of it on two occasions. The Archduke Joseph of Austria also underwent the cure, and it was an amusing sight to see some of the notables of Europe walking barefoot in the dewy grass in frock coats and white cravats. This barefoot walking became the best known system introduced by Father Kneipp. His belief was that most illness was the result of the luxury of modern living and his aim was to improve the circulation and tone up the system. He made use of local bathing and applications together with steam baths which were sometimes medicated with herbs. To stimulate and restore the circulation, he ordered the barefoot walking and cold douches. He always made it a point to see his patients himself, and he made no charges for his services. Contributions from relieved patients he used for parish work. For a long time there were not accomodations for the visitors in the village; but this has been remedied. In recognition of his work, the Pope bestowed on Father Kneipp an honorary office which carried with it the title of Monsignor. In 1894 Monsignor was called to Rome to treat the Pontiff, and it was announced after some time that, by his treatment, the Pope's health had been restored. Kneipp societies have been established in most countries of the world. The method of treatment has made some headway in the United States.—*Scientific American.*

We remember hearing Pfarrer Kneipp deliver an address in a public hall at Heidelberg some five or six years ago. He was then a stout old man with white hair, dark complexion, and bushy, black eye brows. He was enthusiastic over his method of treating disease, which was a crude system of hydrotherapy, and made the most extravagant claims for it. His talk, which, in its quaint homeliness, reminded one of Sam Jones, was not without a strong strain of common sense, particularly in matters of personal hygiene and sanitation, diet, infant feeding. Whatever may have been his faults, Father Kneipp was no quack. His faith was strong and he believed that he was working in the cause of humanity.

